# Transcriptomics and molecular evolutionary rate analysis of the bladderwort (*Utricularia*), a carnivorous plant with a minimal genome

**DOI:** 10.1186/1471-2229-11-101

**Published:** 2011-06-03

**Authors:** Enrique Ibarra-Laclette, Victor A Albert, Claudia A Pérez-Torres, Flor Zamudio-Hernández, María de J Ortega-Estrada, Alfredo Herrera-Estrella, Luis Herrera-Estrella

**Affiliations:** 1 Laboratorio Nacional de Genómica para la Biodiversidad, Centro de Investigación y de Estudios Avanzados del Instituto Politécnico Nacional, 36821 Irapuato, Guanajuato, México; 2 Department of Biological Sciences, University at Buffalo, Buffalo, New York 14260, USA

## Abstract

**Background:**

The carnivorous plant *Utricularia gibba *(bladderwort) is remarkable in having a minute genome, which at ca. 80 megabases is approximately half that of *Arabidopsis*. Bladderworts show an incredible diversity of forms surrounding a defined theme: tiny, bladder-like suction traps on terrestrial, epiphytic, or aquatic plants with a diversity of unusual vegetative forms. *Utricularia *plants, which are rootless, are also anomalous in physiological features (respiration and carbon distribution), and highly enhanced molecular evolutionary rates in chloroplast, mitochondrial and nuclear ribosomal sequences. Despite great interest in the genus, no genomic resources exist for *Utricularia*, and the substitution rate increase has received limited study.

**Results:**

Here we describe the sequencing and analysis of the *Utricularia gibba *transcriptome. Three different organs were surveyed, the traps, the vegetative shoot bodies, and the inflorescence stems. We also examined the bladderwort transcriptome under diverse stress conditions. We detail aspects of functional classification, tissue similarity, nitrogen and phosphorus metabolism, respiration, DNA repair, and detoxification of reactive oxygen species (ROS). Long contigs of plastid and mitochondrial genomes, as well as sequences for 100 individual nuclear genes, were compared with those of other plants to better establish information on molecular evolutionary rates.

**Conclusion:**

The *Utricularia *transcriptome provides a detailed genomic window into processes occurring in a carnivorous plant. It contains a deep representation of the complex metabolic pathways that characterize a putative minimal plant genome, permitting its use as a source of genomic information to explore the structural, functional, and evolutionary diversity of the genus. Vegetative shoots and traps are the most similar organs by functional classification of their transcriptome, the traps expressing hydrolytic enzymes for prey digestion that were previously thought to be encoded by bacteria. Supporting physiological data, global gene expression analysis shows that traps significantly over-express genes involved in respiration and that phosphate uptake might occur mainly in traps, whereas nitrogen uptake could in part take place in vegetative parts. Expression of DNA repair and ROS detoxification enzymes may be indicative of a response to increased respiration. Finally, evidence from the bladderwort transcriptome, direct measurement of ROS *in situ*, and cross-species comparisons of organellar genomes and multiple nuclear genes supports the hypothesis that increased nucleotide substitution rates throughout the plant may be due to the mutagenic action of amplified ROS production.

## Background

The carnivorous plant *Utricularia *and its sister genus *Genlisea *(Lentibulariaceae) share two anomalous molecular evolutionary features: highly increased rates of nucleotide substitution across the genomes of all three cellular compartments, mitochondrial, plastid, and nuclear [1-4], and dynamic evolution of genome size at the level of species or even population [5,6]. Some species, such as *Utricularia gibba *and *Genlisea aurea*, possess the smallest haploid angiosperm genomes known, at ca. 80 and 60 megabases (Mb), respectively, one-half or even less than that of *Arabidopsis thaliana *(*Arabidopsis*), and have bacterial-size chromosomes that vary widely in number between species [5]. Paradoxically, *Genlisea *also contains species with genomes up to 1500 Mb in size. Along with their many physiological and morphological peculiarities, these plants are prime candidates for further research on the complexities of plant physiology associated with carnivory, metagenomic surveys of trap microbial communities, novel plant nitrogen/nutrient utilization pathways, the ecology of prey attraction, whole-plant and trap comparative development, and finally, evolution of the minimal angiosperm genome [6].

With a total of 214 species worldwide, *Utricularia *is the largest genus of carnivorous plants [7]. The name "bladderwort" refers to the bladder-like suction traps that serve for prey capture. Bladders take on many forms within a theme, and their morphologies among species match well with phylogenetic groupings [1]. Additionally, bladders can appear on almost every surface of the plants' leafy or non-leafy structures, as well as in place of a first embryonic leaf [7,8]. Ecologically, the genus comprises predominantly small annual or perennial herbs that occur in three life forms: about 60% of the species are terrestrial, 15% aquatic, and the remaining 25% comprise lithophytes and epiphytes [7]. Like other carnivorous plants, *Utricularia *are typically inhabitants of nutrient-poor environments, and supplement normal photolithotrophic nutrition by trapping and utilizing prey, typically aquatic crustaceans, mites, rotifers and protozoa [9,10]. Previous studies have confirmed nutrient uptake from artificially fed prey in *Utricularia *[11,12], and it is known that organic carbon (C), nitrogen (N) and phosphorus (P) are prominent targets of prey digestion in carnivorous plants [13]. In contrast with other carnivorous plants that acquire carbon from their prey, in some *Utricularia *species photosynthetically absorbed C is secreted into the trap environment [14], suggesting that C supplied into the traps benefits the large associated microbial community, while N and P derived from this community become available for plant uptake in a manner similar to the rhizosphere interactions of terrestrial plants [14,15]. Near zero O_2 _in traps of aquatic *Utricularia *species probably determines the type of organisms that can live inside traps, where a captured prey dies of oxygen deprivation [16]. Digestive extracellular enzymes have been detected on the various trap glands and in the trap fluid [17,18]. It has been proposed that a considerable proportion of enzymatic activity in trap fluid is derived from the commensal organisms that live in *Utricularia *bladders [15]. However, determination of enzyme activities does not prove their origin, with some of them possibly encoded in the *Utricularia *genome.

Despite considerable interest in the biology of Lentibulariaceae, no genomic data is available for these carnivorous plants. Massive parallel 454 pyrosequencing has become a feasible method for de novo transcriptome sequencing with sufficient depth and coverage to carry out quantitative differential gene expression analysis [19-21], which has already been efficiently used for large-scale transcriptome sequencing of different plant species [22-24]. With the aim of determining the *Utricularia *transcriptome and report a detailed analysis of the resulting sequences, we sequenced and assembled 185.5 Mpb of *Utricularia gibba *ESTs. *Utricularia gibba *(Lentibulariaceae) is a free-floating, submerged aquatic carnivorous plant with a small genome of about 80 Mbp [5]. This work provides the first broad survey of nuclear genes transcripts in *Utricularia *species, permitting several hypotheses about their physiology and morphology to be assessed. We detail aspects of the *U. gibba *transcriptome in different organs as well as in plants under physiological stress. Particular attention is paid to the expression of genes involved in N and P uptake, hydrolase-related genes expressed during prey digestion, as well as genes involved in respiration and Reactive Oxygen Species (ROS) production and scavenging. We also report preliminary sequencing of the chloroplast and mitochondrial genomes and provide analyses of molecular evolutionary rates. Finally, using molecular evolutionary analyses and direct experimental methods, we evaluate the hypothesis of Albert *et al.*, 2010 [6], which postulates that Reactive Oxygen Species (ROS) derived from specialized action of cytochrome *c *oxidase account for increased substitution rates and genome-size dynamism following DNA repair.

## Results and Discussion

### Basic analysis of the *Utricularia gibba *transcriptome

Three cDNA libraries were generated from RNA extracted from different organs of *U. gibba *plants [traps: TrpL, shoots: ShtL (vegetative organs), and inflorescences: FlwL (reproductive organs)]. Additionally, a cDNA library from whole plants subjected to multiple physiological stress conditions was generated (StsL) (see Methods for more details). cDNA libraries were sequenced in two 454 pyrosequencing runs. 817,792 masked reads were entered into the assembly process (for more information about masked reads and assembly process see Methods). Using Newbler Assembler software (v2.5; cDNA pipeline) a high proportion of non-assembled reads (singlets) was obtained; this fraction represents approximately one quarter of total masked reads (data not shown). Using a different assembly approach that consisted of clustering/assembling procedures, the vast majority of the masked reads (88.27%) were merged into contigs. The total number of clusters generated was 13,122 that assembled into 16,551 contigs, with an average of 66.4 reads per contig. The length of contigs ranged from 0.1 to 3.0 kb, with an average length of 707.45 bp, suggesting that a significant number of contigs may represent full-length cDNAs. The presence of multiple contigs in a cluster could be due to possible alternative transcripts, paralogy or domain sharing. All reads that did not meet the match criteria to be clustered/assembled with any other reads during the clustering/assembling process were defined as singlets. The total number of singlets was 95,873 (only 11.72% of total masked reads) with an average length of 215.71 nucleotides. Unique transcripts (UT) from *U. gibba *were generated by combining 16,551 assembled contigs and 95,873 singlets.

*U. gibba *UT were annotated by searching for sequence similarities using BLASTX against proteins identified in several available complete plant genomes [*Arabidopsis thaliana, Populus trichocarpa, Ricinus communis, Vitis vinifera *(dicotyledoneous plants), *Oryza sativa, Sorghum bicolor *(monocotyledoneous plants), *Physcomitrella patens *(moss), *Chlamydomonas reinhardtii*, and *Ostreococcus lucimarinas *(green algae), all of them downloaded from the RefSeq database [25]. Using a cut-off e-value of ≤ 10^-05 ^and a bit score ≥ 45 we found that 60,595 (54%) of *U. gibba *UT have high identity to at least one plant protein. The high proportion of *U. gibba *UT with no significant hit (~46%) was expected since the likelihood of finding similarity to previously described proteins is highly dependent on the length of the query sequence. This is illustrated by contig versus singlet hits to database proteins; contigs were found to have significant similarity to plant proteins in over 90% of cases, whereas the majority (55%) of singlets bore no similarity to any proteins. It is also possible that many *U. gibba *UT could not be reliably annotated because they represent untranslated regions (UTRs) or non-coding RNAs (ncRNAs). A comparison of *U. gibba *UT against the *U. gibba *genome sequence (assembled using Celera; [26,27]) using BLASTN shows that 85.2% of the transcripts have a significant hit against the genome (98% of alignment length and minimal sequence identity of 90% over the complete alignment). The remaining sequences probably failed to align because the *U. gibba *genome is currently represented by a preliminary draft assembly of relatively low coverage (~8x, E. Ibarra-Laclette et al., unpublished data).

We determined the proportion of plant proteins for which homology was detected among *U. gibba *UT. Homology was detected to 43% of *Arabidopsis *(14,382 of 33,405), 38% of *Populus *(16,202 of 42,344), 40% of *Ricinus *(12,494 of 31,221), 55% of *Vitis *(13,017 of 23,493), 47% of *Oryza *(12,652 of 26,940), 38% of *Sorghum *(12,472 of 33,005), 30% of *Physcomitrella *(10,789 of 35,936), 30% of *Chlamydomonas *(4,441 of 14,503) and 47% of *Ostreococcus *proteins (3,621 of 7,603). *U. gibba *UT were similar, at most, to 16,202 unique plant proteins (Additional file 1, Table S1). This number represents the most stringent underestimation of the minimal number of *U. gibba *genes found expressed in the organs and conditions sampled in this study.

**Figure 1:**
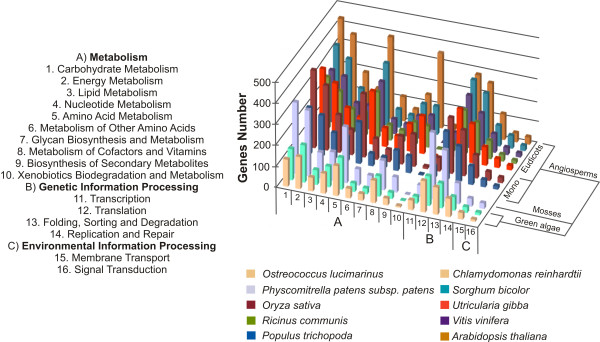
**Functional annotation**. Proportion of KEGG categories (Kyoto Encyclopedia of Genes and Genomes) found in the *U. gibba *unique transcripts (UT) compared with plants genome annotations [(*Arabidopsis thaliana, Populus trichocarpa, Ricinus communis, Vitis vinifera *(dicotyledon plants), *Oryza sativa, Sorghum bicolor *(monocotyledon plants), *Physomitrella patens *(moss), *Chlamydomonas reinhardtii*, and *Ostreococcus Lucimarinuas *(green algae's)].

The Kyoto Encyclopedia of Genes and Genomes (KEGG) classifications [28] from best-hit plant proteins were associated to *U. gibba *UT in order to identify proteins with a known function. Proportions of best hits in each KEGG category are shown in Figure 1. Additionally, using the KEGG Atlas resource [29] we created a global metabolism map combining 119 existing pathways, corresponding to 16,595 genes referenced to in the KEGG database for *Arabidopsis, Populus, Vitis, Ricinus, Oryza, Sorghum, Physcomitrella, Chlamydomonas *and *Ostreococcus*. This global metabolism map was compared to the global map created for the *U. gibba *UT, for which 117 distinct metabolic pathways could be assigned (Additional file 2, Figure S1) out of 119 plant metabolic pathways annotated in the KEGG Atlas. These results indicate that the *U. gibba *UT comprise a deep representation of the complex metabolic pathways that characterize a plant genome, permitting their use as a source of genomic information to explore the structural, functional, and evolutionary diversity of the Lentibulariaceae.

### Identification of *U. gibba *transcription factor (TF) families

Plants devote ~7% of their genome coding capacity to proteins that regulate transcriptional activities [30-32]. Analysis of completed plant genome sequences suggests that over 60 transcription factor (TF) families are present in most plant genomes. In *Arabidopsis *[33,34] and *Populus trichocarpa *[35,36] the 64 TF families vary in size from 1-2 members to over 100 members. Rice contains 63 of the 64 dicot TF families [38,39], missing only the SAP1 family, which is represented by a single gene in both *Arabidopsis *and *P. trichocarpa*. About ~3% (3,222) of the *U. gibba *UT showed significant homology (BLASTx; e-value ≤ 10^-05 ^and a bit score ≥ 45) to known TFs previously defined in *Arabidopsis *[33,34] and were similar to a maximum of 920 unique TFs. We examined the distribution among the known TF families in vascular plants, and in selected cases, the complexity of *U. gibba *TF families relative to what is found in other plant species. At least one member for 61 of the 64 TF families previously identified in vascular plants was identified in *U. gibba *UT. Among the low copy TF families present in other plants, one member of each of the HRT-like, LFY, Whirly, S1Fa-like and VOZ families, two members of the BBR-BPC, CCAAT-DR1, CPP, GIF and MBF1 gene families, and 3 members of the C2C2-YABBY and EIL gene families are represented in the *U. gibba *UT. Only the SAP1, NZZ and ULT TF families were not represented among the *U. gibba *UT (Additional file 3, Table S2).

Since *U. gibba *is a plant that lacks roots, it was possible that genes involved in root development had been lost, contributing to a reduction in genome size. Although the transcriptomes would never be a full representation of all genes present in a given genome, interestingly, we found that most of the TFs preferentially expressed in and known to be involved in root development, including homologous proteins to the *A. thaliana *ARFs 5, 7, 19, AUX/IAA proteins 3, 7, 12, 14 and 17; Short Root and Scarecrow (members of GRAS family) are represented in the *U. gibba *transcriptome [37]. This finding suggests the possibility that the lack of roots in *U. gibba *may not be due to a preferential loss of genes involved in root development but instead a loss of developmental programs involved in the establishment of the gene expression networks required for root formation.

### Changes in transcript abundance in the *U. gibba *transcriptome

Each organ-specific transcriptome was significantly sampled, and only a low disparity among the number of reads in each organ was detected (258,457 reads for FlwL, 234,963 for ShtL, and 292,970 for TrpL). The transcriptome obtained from *U. gibba *plants exposed to different stresses (pooled from constant light, darkness, cold temperature, and drought conditions) was also included in our analysis (represented by StsL, 140,507 reads). A large proportion of the reads (88.27%) assembled into 16,551 contigs, each assumed to represent a distinct gene structure. In principle, the number of reads that assemble in a specific contig represents the abundance of mRNA produced by a particular gene in a given tissue sample. However, differences in transcript abundance may reflect sampling errors rather than genuine differences in gene expression. In consequence, read counts must be normalized to allow comparison of expression measures across samples, and a common practice is to scale gene counts by library totals [38,39]. Recently, however, it has been reported that more general quantile-based procedures yield much better concordance with expression pattern values obtained by qRT-PCR [40]. Therefore, we decided to normalize read-counts in the R environment [41] using a quantile normalization procedure similar to that described previously by Bullard *et al. *2010 [40], which is based on a previously described microarray normalization approach [42]. An expression profile matrix was created (Additional file 4, Table S3) containing the number of reads for each of the 16,551 genes represented by contigs (rows) and four normalized transcriptomes (columns). Normalized read counts ranged from 0-3500.

**Figure 2:**
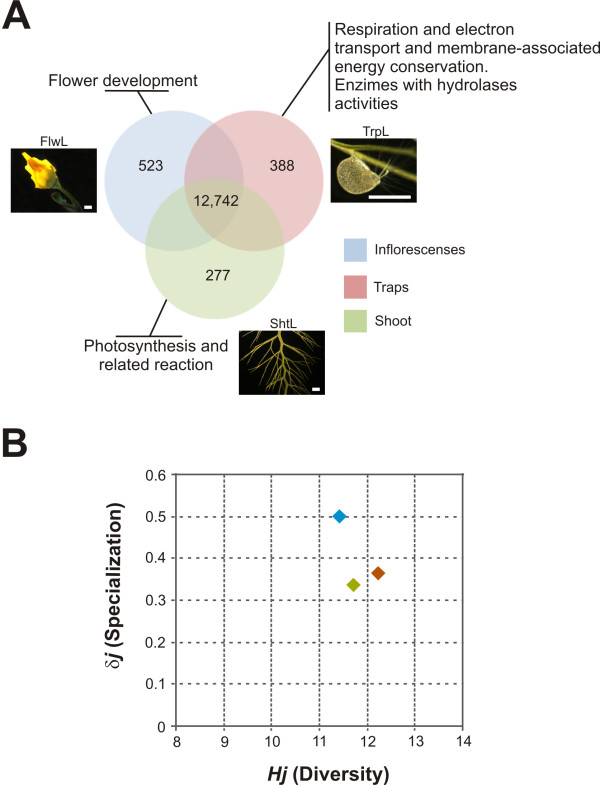
**Analysis of the *Utricularia gibba *transcriptome**. **(A) **Venn diagram of ubiquitously and preferentially expressed genes (PEG). Biological processes over-represented by PEG are summarized in figure. **(B) **Scatter plot of the values of diversity, *Hj *vs. the values of specialization given by the average gene specificity of the organs, *δj*.

To assess the relative abundance of gene transcripts among organ-specific transcriptomes, we applied the statistical R test [43]. We considered preferentially expressed genes (PEGs) to be contigs with R ≥ 8 (true positive rate of ~98%) and a 2-fold minimum difference in terms of reads per organ-specific transcriptome as compared against the other sequence sets. A total of 1,181 *U. gibba *UT were identified as PEGs; 523 in FlwL, 277 in ShtL and 388 in TrpL, some of which could be considered as organ-specific genes because of all reads forming these *U. gibba *contigs were derived from a single cDNA tissue sample (Figure 2A and Additional file 5, Table S4). To identify ubiquitously expressed genes we considered only those clusters with at least one read from every library. In this case, all statistical tests were required to have non-significant results (Additional file 6, Table 5). Stress responsive genes were identified by comparing the transcriptome obtained from *U. gibba *stressed plants (represented by StsL) against all organ-specific data sets. According to the stringency levels (R ≥ 8 and fold ± 2) a total of 200 *U. gibba *UT were identified as differentially expressed genes in response to multiple physiological stresses (Additional file 7, Table S6).

In order to quantify the similarity among organ-specific *U. gibba *transcriptomes we compared their diversity and specialization using a recently described model based on Shannon entropy. Diversity (*Hj*) is measured by an adaptation of Shannon's formula for entropy of a transcriptome's frequency distribution, while specialization (*δj*) is estimated as the average specificity of the genes expressed in each organ [44]. The estimation of these properties allows the recognition of general differences among the transcriptomes, enhancing the understanding of their distributions. We note that the most specialized organ sampled in *U. gibba *is the inflorescence, even when the traps, characteristic for the genus, are among the most intricate structures in the plant kingdom and are the organ through which *Utricularia *attract, capture and digest their prey [10,45,46]. The diversity measures of the three organ classes (shoot, inflorescences and traps) group in a region of relatively low diversity (Figure 2B). Shoots and traps, however, could be considered as extremely similar organs based on their transcriptomes. This is not surprising, however, given that bladders are in fact modified leaves with sensitive bristles on "trap door" entrances [45].

### Functional classification of differentially expressed genes highlights energy, metabolism, and hydrolases

PEGs in a specific organ were classified into functional categories according to the Munich Information Center for Protein Sequences classification (MIPS) using the FunCat database [47,48] and an *Arabidopsis *annotation was obtained for *U. gibba *UT (Additional file 8, Table S7). A Venn diagram was constructed to show selected overrepresented categories and their intersections in inflorescences, traps and shoots (Figure 2A). As one validation of differential expression in these tissues, among inflorescence PEGs, the MIPS category "Tissue differentiation" was significantly over-represented via the subcategory 'flower' (Supplementary Table 6). Furthermore, 15 genes for which expression was considered as PEG among the transcriptomes were selected with the aim of validating expression patterns found. In general we found a good correlation (r^2 ^= 0.89) of the expression levels obtained by 454 sequencing with those obtained by qRT-PCR (Additional file 9, Figure S2).

A noteworthy over-represented MIPS category identified in shoot and trap PEG was "Energy". In shoot PEG the "Energy" MIPS category is represented by 'photosynthesis' and 'energy conversion and regeneration' subcategories, while in trap PEG, this category is represented by 'respiration' and 'electron transport and membrane-associated energy conservation' subcategories (Additional file 8, Table S7). As expected in shoot PEG, the *U. gibba *UT annotated as SBPase (Sedoheptulose-biphosphatase; AT3G55800) and RuBisCo small subunit 1B (AT5G38430) were identified as over-represented in the "Metabolism" MIPS category (represented by subcategory 'autotrophic CO_2_-fixations') (Additional file 8, Table S7). These results suggest that whereas photosynthesis occurs mainly in the shoot, in traps respiration is the major metabolic activity.

With regard to PEG in traps, some *U. gibba *UT were annotated as hydrolases (Additional file 5, Table S4). These *U. gibba *UT were: CL12267contig15708 (putative aminopeptidase; similar to AT4G30920), CL3763contig07204 (putative α-glucosidase; similar to AT5G11720), CL434contig01978 (putative β-glucosidase; similar to AT1G02850), CL6134contig09575 (putative β-hexosaminidase; similar to AT1G65590) and CL851contig02926 (putative purple acid phosphatase; similar to AT1G14700). Activities for the same five hydrolases have been reported in the fluid collected from traps of four aquatic *Utricularia *species (*U. foliosa, U. australis, U. aurea *and *U. vulgaris*) [17,18].

### Nitrogen and phosphorous uptake in *U. gibba*

Nitrogen and phosphorous are two essential macronutrient elements for plants, that are often a major constraint for plant growth and reproduction in both terrestrial and aquatic ecosystems. The major forms of these nutrients utilized by plants are nitrate (NO_3_^-^) and phosphate (H_2_PO_4_^-^; Pi). A number of genes encoding the transporters and channels for nutrient acquisition have been identified and functionally characterized in model species, particularly *Arabidopsis *and rice [49-51]. It has been proposed that phosphorus uptake from prey might be more important than that of nitrogen [17]. Trap fluid stoichiometry (molar N:P ratios about 100) as well as the presence of nutrient limited microbial cells (molar N:P ratios 25-61) indicates the importance of phosphorus rather than nitrogen for the nutrition of *Utricularia *[15]. Additionally, in *U. vulgaris *it has been reported that investment in carnivory, calculated as the proportion of leaf biomass and leaf area comprising traps, is inversely proportional to the availability of Pi from non-carnivorous sources, whereas N showed no significant effect in the investment in carnivory [52]. This is consistent with the notion that phosphorus uptake from prey might be more important than that of nitrogen for *Utricularia *species. A gene encoding an acid phosphatase is the highest expressed among *Utricularia *PEGs (Additional file 9, Figure S2), and genes encoding three members of the Pht1 family of high affinity Pi transporter were identified as PEGs in traps (Additional file 10, Table S8). Since the Pht1 family comprises high-affinity Pi strongly expressed in plant roots [53-58], we suggest that in rootless *Utricularia *Pi uptake takes place mainly in the traps [8,59].

In higher plants there are two types of nitrate transporters, named NRT1 and NRT2s (low- and high-affinity nitrate transporters) [60]. Microarray experiments have been used to identify additional genes involved in nitrate/nitrite assimilation [61]. Using this information we identified a total of 77 *U. gibba *UT annotated as homologous to *Arabidopsis *proteins involved in the nitrate assimilation pathway (45 members from the NTR1 family, 3 from the NTR2 and 23 Nitrate/nitrite-assimilation genes) (Additional file 11, Table S19). Most of these genes were found to be ubiquitously expressed in *U. gibba*, with the exception of the homolog of the *Arabidopsis *CHL1 gene that was identified among the shoot PEGs. CHL1 (AT1G08090) is a NTR2 protein that recently has been reported to function as a nitrate sensor in plants [62]. Additionally we found that three different *U. gibba *UT annotated as δ-TIP (Tonoplast Intrinsic Protein; AT3G16240) were among the most highly expressed genes in shoot. δ-TIP (AT3G16240) has recently been reported as an ammonium (NH_4_) transporter, since δ- and γ- TIP's (AT3G16240 and AT2G36830, respectively) complement the lack of urea transporters in yeast [63]. In the bladderwort *Utricularia vulgaris*, 51.8% of the total nitrogen content has been estimated to come from insect derived nitrogen [12], however, contribution of nitrogen from animal prey is variable in carnivorous plants, with estimates ranging from 10% to 87% dependent on taxa [64]. Considering the high amino acid identity (Additional file 12, Figure S3), ranging from 59.2 to 78.9% among the *Utricularia *and *Arabidopsis *Tonoplast Intrinsic Proteins (TIPs), these results suggest that in aquatic *Utricularia *species, nitrogen uptake, at least in part, could be taking place in shoot (stem/leaves) and that urea could be a major N source for aquatic *Utricularia *species.

### Elevated molecular evolutionary rates in organellar genome blocks and individual nuclear genes

In addition to transcriptome discovery, we sequenced large portions of the plastid and mitochondrial genomes from *Utricularia gibba *as part of our *Utricularia *nuclear genome sequencing project. This has provided us with an unprecedented opportunity to evaluate earlier findings on elevated molecular evolutionary rates in *Utricularia *organellar genomes [1-4]. From 2.2 million *U. gibba *whole-genome shotgun (WGS) sequencing reads (748 Mbp, representing more than 8 times the estimated genome size) 76,364 high-quality reads were identified as organellar sequences (27.6 Mbp). These reads were assembled using Newbler assembler version 2.5, resulting in 228 contigs from chloroplast and 217 contigs from mitochondrial genomes with a N50 contig size of 2,146 and 2,842 bp respectively. The largest *U. gibba *chloroplast contig (length = 22,577 bases; FTP: http://www.langebio.cinvestav.mx/utricularia/) corresponds to part of the large single copy region (LSC; [69,70]). Using a Multiple Genome Comparison and Aligment Tool [65,66] we selected a homologous region from 31 of 64 eudicot (Rosids and Asterids) angiosperm chloroplast genomes contained in an organelle genome database [67,68], this chloroplast region encodes a total of 28 coding genes. Removal of ambiguously aligned was carried out using GBlocks [69], which is designed to identify and remove highly variable regions of alignments where positional homology is dubious (Additional file 13, Figure S4). The final ClustalW alignment [70] contained 31 taxa and 8,516 nucleotide characters for the fraction of the LSC chloroplast region. For the mitochondrial genome we made a similar analysis as described above for the chloroplast sequences using unambiguously aligned sequences (length = 4,125 bases; FTP: http://www.langebio.cinvestav.mx/utricularia/) derived from a mitochondrial contig of 4,673 nucleotides (Additional file 14, Figure S5). A total of four coding genes were identified in this partial sequence of the *U. gibba *mitochondrial genome. Due to the limited number of complete sequences of mitochondrial genomes, phylogenetic analysis was carried out using the homologous region from six eudicot taxa.

**Figure 3:**
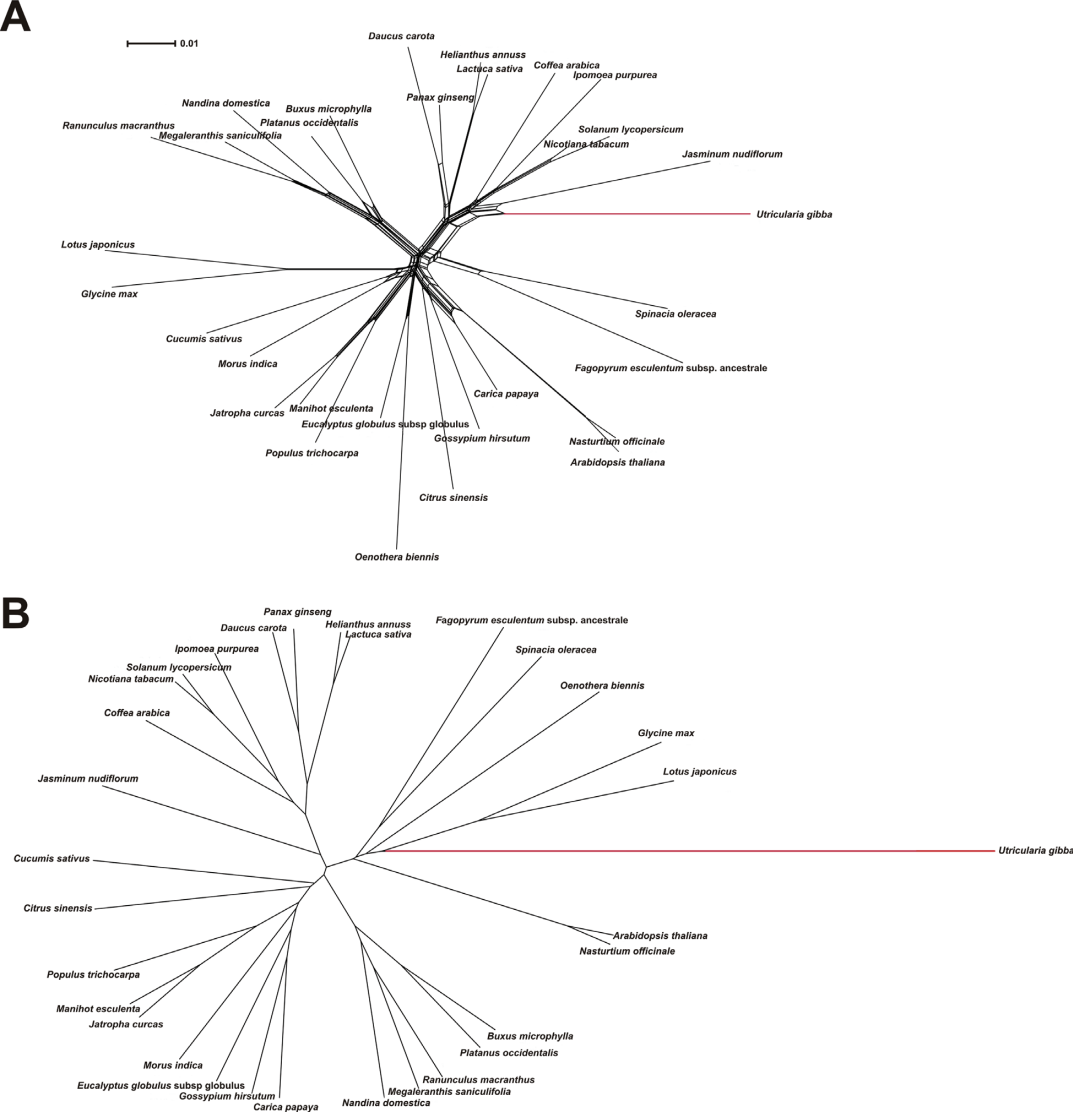
**A long contig of the plastid genome shows an elevated substitution rate in *Utricularia gibba***. Although this phenomenon is only slightly observable in NeighborNet phylogenetic analysis (A), it is remarkable in a UPGMA phenogram (B), which assumes clock-like rates. The data analyzed are for eudicots only.

**Figure 4:**
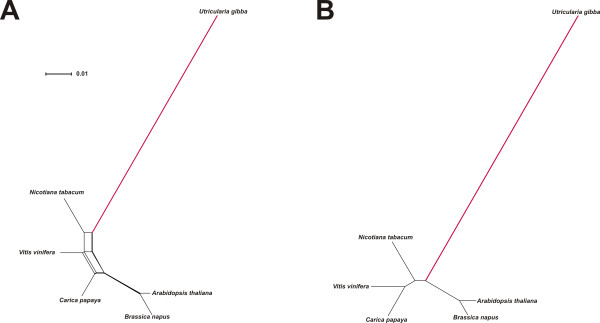
**A portion of the mitochondrial genome shows a dramatically elevated nucleotide substitution rate in *Utricularia gibba***. Both the NeighborNet phylogenetic analysis (A) and UPGMA phenogram (B) show Utricularia on a very long external branch.

NeighborNet phylogenetic analysis [71] was used as a simple tool to illustrate both branch length differences among species and incongruence of phylogenetic signal within data sets. Analysis of the large block of chloroplast LSC sequence revealed that *Utricularia gibba *has the longest terminal branch of any eudicot sampled (Figure 3A). Although this relative rate difference is slight, it is statistically significant at *P *< 0.05 (using several likelihood models; see Methods) with respect to *Jasminum *(jasmine), the sister genus of *U. gibba*, as analyzed using *Coffea *(coffee) as outgroup (Figure 3A). Elevated evolutionary rate in *U. gibba *is, however, striking in a rate-sensitive UPGMA cluster analysis [72] of the same data (Figure 3B). UPGMA assumes a molecular clock operating equally among all species, so deviation from this requirement in terms of obtained branch lengths, and possibly also well-established phylogenetic relationships, provides a useful test for rate asymmetries. Accordingly, the plastid DNA UPGMA tree places *U. gibba *erroneously, separate from asterid taxa to which this species is assuredly most closely related (Figure 3B). For the mitochondrial genome, NeighborNet analysis (Figure 4A), relative rate tests (*Utricularia *vs. *Nicotiana*, outgroup *Vitis*; *P *<< 0.001 across several tests), and UPGMA clustering (Figure 4B) of the available data all demonstrate an enormously elevated substitution rate in *Utricularia*.

**Figure 5:**
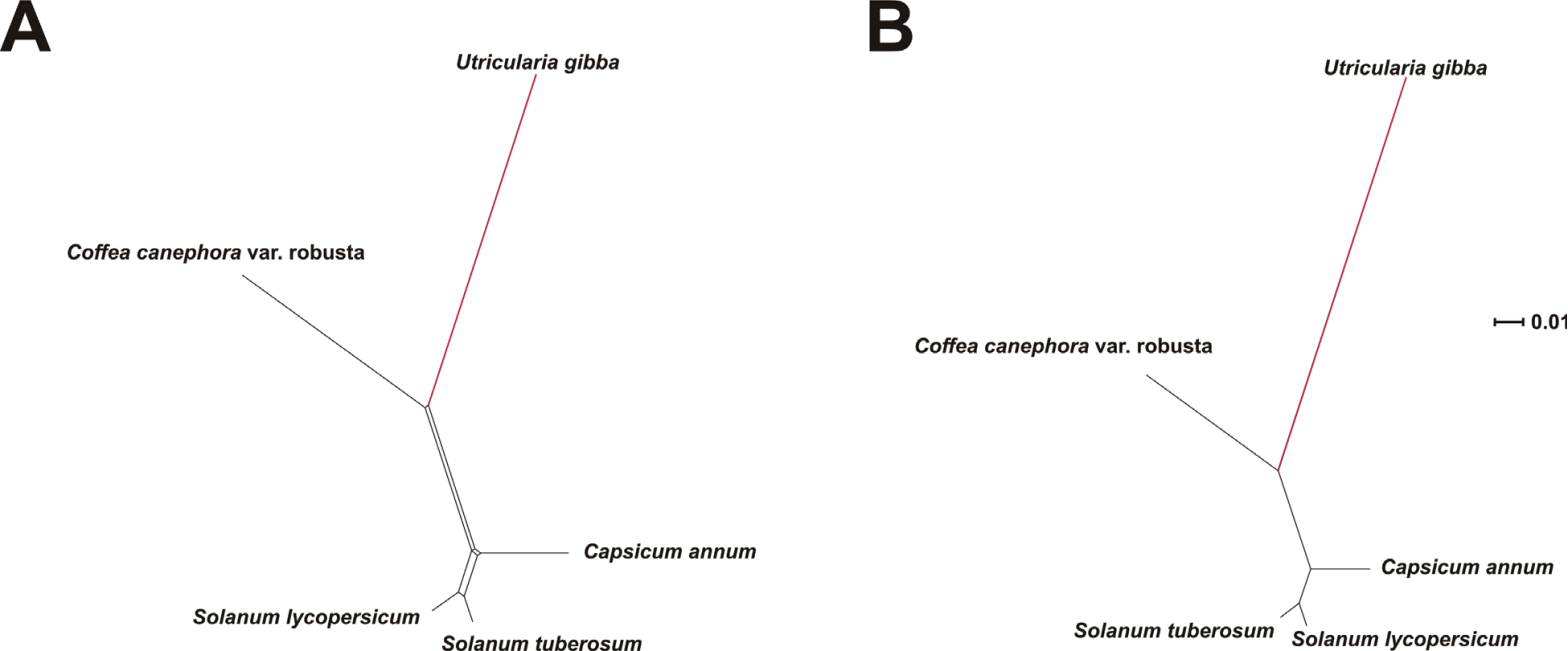
**A super-matrix of 100 distinct nuclear gene alignments from the Conserved Ortholog Set (COS) database demonstrates *Utricularia gibba *to have the highest relative substitution rate among analyzed asterid species**. Both NeighborNet analysis (A) and a UPGMA test (B) clearly show this asymmetry.

Given the availability of considerable nuclear transcriptome sequence, we also assayed molecular evolutionary rates across a random set of 100 genes homologous to Conserved Orthologous Loci (COS II) available for several other asterid species [73-75]. Here, we found that *U. gibba *displayed the longest branch in NeighborNet analysis - and therefore the highest relative molecular evolutionary rate - for 92% of these loci. Consistently, UPGMA analyses identified the *U. gibba *branch as longest in 90% of the 100 loci (all 100 data sets, networks and trees are available via FTP: http://www.langebio.cinvestav.mx/utricularia/). A concatenated super-matrix comprising all gene sequences for all species produced expected NeighborNet (Figure 5A) and UPGMA (Figure 5B) results, with *U. gibba *displaying an elevated molecular evolutionary rate that was significant at *P *<< 0.001 with respect to *Coffea arabica *(outgroup *Capsicum annuum*, using the same likelihood models as for the organellar genomes).

### Carbon, respiration, and Reactive Oxygen Species

Analysis of the *U. gibba *choloroplast and mitochondrial genomes shows that nucleotide substitution rates are elevated in *U. gibba*. These alterations in substitution rates have been proposed to be related to specific changes in oxidative phosphorylation and excess production of reactive oxygen species (ROS; see below). Therefore, we analyzed the functional categorization of shoot and trap PEG to determine whether they provide molecular support for ox-phos and ROS related processes. As previously mentioned, among the prominent over-represented MIPS category identified in shoot and trap PEG was "Energy". In shoot PEG the "Energy" MIPS category is represented by 'photosynthesis' and 'energy conversion and regeneration' subcategories, while in trap PEG, the "Energy" category is represented by 'respiration' and 'electron transport and membrane-associated energy conservation' subcategories. Correspondingly, *Utricularia *bladders have immensely greater respiration, while exhibiting far lower photosynthetic rates than vegetative tissues [76,77]. Interesting in connection, the 'oxygen and radical detoxification' subcategory was prominent among stress PEG.

The respiratory chain of mitochondria, normally coupled to electron transport, is one of the main means by which cells gain their energy for performing various activities. Electron transport drives a chemiosmotic pump that causes sequestration of protons in the mitochondrial intermembrane space, where after these positive charges enter the mitochondrial lumen to catalyze the phosphorylation of adenosine diphosphate into ATP. The rate limiting enzyme of oxidative phosphorylation is cytochrome c oxidase (COX), positioned one step before ATP synthase. Previous reports showed that, due to changes in specific amino acid positions fixed under positive Darwinian selection, COX structure and function might be altered in *Utricularia *and some species of its sister genus, *Genlisea *(the corkscrew plant). Hypotheses have been proposed whereby specific changes in these residues [two contiguous cysteines (C)] could alter the dissociation kinetics between COX and cytochrome c [78] and possibly produce a conformational change at the active site [79]. It has been suggested that the latter process could reversibly decouple proton pumping from electron transport [79]. In this way, the intermembrane space could be likened to a capacitor holding enormous positive charge until ATP was needed, e.g., to pump water out of traps after their firing. However, storing large quantities of protons could have consequences in the formation of reactive oxygen species (ROS) that could be produced by back-up and leakage of electron transport [6]. It then follows that the mutagenic action of enhanced ROS production (with error-prone repair) may, as a common cause, explain both the high rates of nucleotide substitution observed in *Utricularia *(above) and the dynamic evolution of genome size in Lentibulariaceae, the latter via non-homologous recombination at double strand breaks [5].

Using the FunCat database and MIPS categorization we identified a total of 18 annotated *U. gibba *UT (homologous to 15 *Arabidopsis *unique proteins) grouped into the "DNA recombination and DNA repair" MIPS subcategory, all of them considered as ubiquitously expressed genes (Additional file 15, Table S10). With regard to the "oxygen and radical detoxification" subcategory, 159 *U. gibba *UT corresponding, at most, to 91 *Arabidopsis *unique proteins, some of which (44.7%) were also expressed ubiquitously (Additional file 16, Table S11). This MIPS subcategory includes 35 proteins involved in glutathione conjugation and peroxidase reactions, 6 proteins involved in superoxide metabolism and one catalase. Again, expression of these DNA repair and ROS detox processes is neither trap-specific nor trap-overexpressed, but ubiquitous. However, ROS production need not be evenly distributed among organs, which could alter net repair/detox capacity in living tissues.

**Figure 6:**
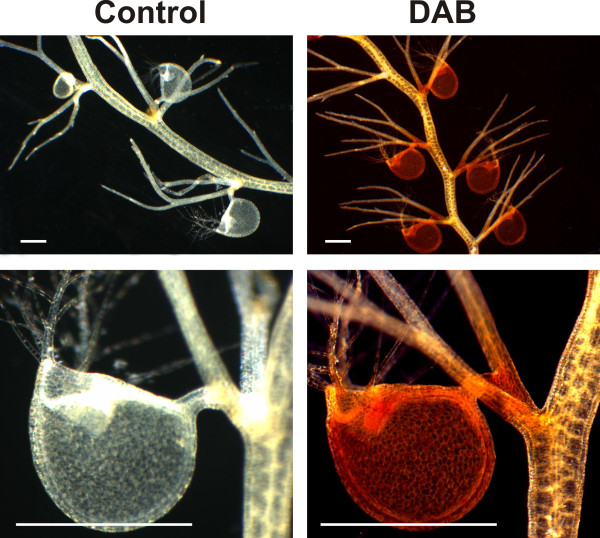
**Detection of Reactive oxygen species (ROS) in *Utricularia***. ROS patterns of *Utriculara gibba *plants stained with H_2_O_2 _specific dye 3, 3-Diaminobenzidine (DAB). Photographs are representative of at least 10 stained plats. Bars = 100 μm.

In order to evaluate ROS content in traps versus vegetative organ cells, *U. gibba *plants were stained with the H_2_O_2 _specific dye 3, 3-Diaminobenzidine (DAB). DAB staining was detected in most cells of vegetative organs (stem/leaves and traps) with a considerably higher intensity in the traps (Figure 6), corresponding well to the much greater respiration observed in these structures by Adamec 2006 [77]. Given the ubiquitous expression patterns in all *Utricularia *organs of genes encoding detoxification enzymes, lower relative ROS detoxification is expected in traps and therefore greater toxic effects such as mutagenesis. It is therefore certainly possible that the observed elevated nucleotide substitution rates in *Utricularia *organellar genomes and nuclear genes are due to ROS overproduction in the face of net less effective DNA repair. Although the null expectation would be that all nuclear genes should accrue ROS-mediated mutations equally and randomly, 8% out of the 100 genes surveyed did not show evidence for an *Utricularia*-specific rate increase. Analytical error and/or differential evolutionary conservation of gene sequences in different species could explain this small disparity from 100% expectation. Future analyses of nonsynonymous vs. synonymous substitution rates in these cases might reveal the latter phenomenon. With regard to nuclear genome dynamism, detailed studies, e.g., searches for molecular signatures of rampant double strand break repair, must await a high-quality sequence of the entire *Utricularia gibba *genome.

## Conclusions

The *Utricularia *transcriptome provides a detailed genomic window into processes occurring in a carnivorous plant. It contains a deep representation of the complex metabolic pathways that characterize a putative minimal plant genome, permitting its use as a source of genomic information to explore the structural, functional, and evolutionary diversity of the genus. Vegetative shoots and traps are the most similar organs by functional classification of their transcriptome, the latter expressing hydrolytic enzymes for prey digestion that were previously thought to be encoded by bacteria. Supporting physiological research, traps significantly overexpress genes involved in respiration. Other expression data suggests that whereas nitrogen uptake could in part take place in vegetative parts, phosphate uptake might occur mainly in traps. Expression of DNA repair and ROS detoxification enzymes may be indicative of response to increased respiration. Finally, evidence from the bladderwort transcriptome, direct measurement of ROS *in situ*, and cross-species comparisons of organellar genomes and multiple nuclear genes supports a hypothesis that increased nucleotide substitution rates throughout the plant may be due to the mutagenic action of amplified ROS production.

## Methods

### Plant material and treatments

*Utricularia gibba *plants were collected at the Umécuaro dam (Michoacán-México), and propagated outdoors in plastic containers (area 0.1 m2, 10 L), filled to a depth of 12.5-15 cm with water from the dam; the water level maintained by addition of soft tap water. Tissue was collected from shoot-like structures, traps and inflorescences containing 1 to 4 flowers.

To obtain evidence for the expression of as many genes as possible, additional sets of *U. gibba *plants (3 to 5 individuals) were transferred into a sterile 50 mL Erlenmeyer flask (containing 25 ml of sterile water) and then exposed to different physiological stress conditions (light, temperature and water deprivation). For light responses, *U. gibba *plants were treated in continuous darkness for 48 hrs and then exposed to constant light (100 μmol m^-2 ^sec^-1^). Samples were collected at 4, 8, 12 and 24 hrs; as a control *U. gibba *plants were kept in the dark and collected at the same time points. For low temperature responses, *U. gibba *plants were transferred to a cold room at 5°C with constant light (approximately 50 μ ;mol m^-2 ^sec^-1^) for 48 hrs. Samples were collected at 4, 8, 12, 24 and 48 hrs. Drought-stress treatments (water deprivation) consisted of removing the water from flasks where after *U. gibba *plants were kept in controlled environment chambers at 24°C under constant light (50 μmol m^-2 ^sec^-1^). Samples were collected at 4, 8, 12, 24 and 48 hrs. As a control for low temperature and drought-stress treatments *U. gibba *plants were kept in water in controlled environment chambers at 24°C under constant light (50 μmol m^-2 ^sec^-1^). Additionally, we collected traps, shoots (considering these as stem with life-like structures) and inflorescences from *U. gibba *plants with the aim of exploring and comparing the transcriptome of these organs. All samples collected were frozen immediately in liquid nitrogen and stored at -70°C until used.

### cDNA library construction

Total RNA was extracted from *U. gibba *organs or whole plants exposed to the different stress conditions using TRIZOL (Invitrogen), ground with a mortar and pestle in liquid nitrogen. In the case of plants subjected to stress, 5 μg of RNA from each experimental condition was pooled to obtain a single RNA sample. First and second strand cDNA synthesis was performed with 3 μg of the total RNA mixture using Message Amp-II kit (Ambion) following the manufacturers recommendations. In the case of the three organs (traps, shoots and inflorescences), each sample was treated separately. 10-12 ng of synthesized cDNA was amplified by *in vitro *transcription and the resulting 70-90 μg of antisense RNA (aRNA) was purified using Qiagen RNAeasy columns (Qiagen). A second round of cDNA synthesis was performed using the mRNA as template (20 μg). cDNA synthesis was performed as described above except that random primers (mostly hexamers) were used at the first strand synthesis stage. This procedure yielded approximately 10 μg of cDNA that was purified using the DNA Clear Kit for cDNA purification (Ambion). cDNA was then treated with Ampure magnetic particles (Agencourt, Beckman Coulter) to obtain fragments of 200 - 700 pb.

### 454 cDNA sequencing and assembly

Approximately 10 μg of sheared cDNA was used for 454 sequencing. The cDNA sample was end repaired and adapter ligated according to the manufacturer's instructions. Streptavidin bead enrichment, DNA denaturation and emulsion PCR were also performed according to described procedures [80]. Typical output from a 4.5-h run of the GSFLX sequencer is around 75 Mb, comprising roughly 300,000 sequence reads averaging *c*. 250 bp. Two FLX pyrosequencing runs were performed (the pico-titer plate was divided in two sectors), 1/2 run from StsL (cDNA pool obtained from *U. gibba *plants exposed to multiple physiological stress condition; stress Library), 1/2 run for ShtL (shoots library), 1/2 run for FlwL Inflorescences library) and 1/2 run for TrpL (utricles or traps Library). A total of 926,897 reads were generated (258,457 for FlwL, 234,963 for ShtL, 140,507 for StsL and 292,970 for TrpL) with an estimated average size of 200.18 bases representing a total of 185.55 Mpb. Reads were masked using the SeqClean software pipeline [83] to eliminate sequence regions that would cause incorrect assembly. Targets for masking include poly A/T tails, ends rich in Ns (undetermined bases) and low complexity sequences. To carry out the assembly process, 817,792 masked reads (88.23% of total, with a minimum size of 100 bp) were considered. Masked read sequences were pairwise compared and grouped into clusters, based on shared sequence similarity. As a consequence, the clusters obtained comprise reads most likely derived from the same mRNA. Each cluster was then assembled into one or more contigs, which were derived from multiple read alignments. The clustering was performed using *megablast *[81] and the resulting clusters were then assembled using the CAP3 assembly program [82]. Contigs within a cluster shared at least 90% identity within a window of 40 nucleotides. The combination of contigs and singletons are referred to as unique transcripts (UT; 16,551 contigs and 95,873 singletons respectively). Files containing sequence reads and quality scores were deposited in the Short Read Archive of the National Center for Biotechnology Information (NCBI) [Accession number SRP005297].

### Annotation of *U. gibba *UT

BLASTx similarity searches (e-value 10^-5^, bit score ≥ 45) against proteins referenced in the RefSeq database [25] from 9 complete plant genomes [*Arabidopsis thaliana, Populus trichocarpa, Ricinus communis, Vitis vinifera *(eudicots), *Oryza sativa, Sorghum bicolor *(monocots), *Physomitrella patens *(moss), *Chlamydomonas reinhardtii*, and *Ostreococcus lucimarinas *(green algae)] were performed to annotate the *U. gibba *UT. We used the *Arabidopsis *protein annotation to define homologous genes in *U. gibba *because these annotations are more refined than those from other species. The UT were also assigned to functional categories using the Kyoto Encyclopedia of Genes and Genomes (KEGG) [83-85] and Enzyme Commission (EC) numbers [86] were associated.

### Expression profile analysis of *U. gibba *transcriptome

Transcripts appearing more than once in the cDNA libraries (FlwL, ShtL, TrpL and StsL) were selected for *in silico *expression analysis after quantile normalization and statistical testing [42,43]. In brief, this method allows the comparison of gene expression among any number of libraries in order to identify differentially expressed genes. The method uses a single statistical test to describe the extent to which a gene is differentially expressed between libraries by a log likelihood ratio statistic that trends asymptotically to a χ^2 ^distribution [43]. Results were visualized using GeneSpring GX 7.3.1 software (Agilent Technologies^®^).

### Phylogenetic, cluster, and molecular rate analyses

Concatenated blocks of plastid and mitochondrial DNA genomes (defined using Gblocks [75]) and nuclear gene sequences were analyzed using NeighborNet [71] and UPGMA [72] in the SplitsTree4 package [87]. The 100 randomly chosen nuclear gene data sets were obtained by alignment (using MUSCLE [88] against translated amino acids) of *Utricularia gibba *cDNA contigs and *Arabidopsis *gene sequences to asterid orthologs available in the COS II database. For phylogenetic analysis, the LogDet distance [89] was used as a standard model-based correction throughout due to its robustness against base composition bias, which we empirically observed using a diagnostic utility in SplitsTree4. Relative substitution rate differences were calculated using HyPhy [90] under several models (F81 with fixed rates, and F84 with fixed rates, general time reversible either with local parameters fixed, with global parameters, or with global parameters, gamma distribution and 4 rate classes). *P *values are reported as less than the largest value obtained under the different models.

### Functional classification of differentially expressed genes

Functional gene classification was performed according to the Functional Catalogue [47,48]. The hypergeometric method with Bonferroni correction was used for the analysis with a P-value cutoff of 0.01.

### qRT-PCR

Primer design (Tm, 60-65°C) was performed in the Primer3 v.0.4.0 web tool [91]. cDNA templates for PCR amplification were prepared using reverse specific primers for each gene evaluated, and treated with SuperScript III reverse transcriptase (Invitrogen) according to the manufacturer's instructions. Each reaction contained cDNA template from 10 μg total RNA, 1× SYBR Green PCR Master Mix (Applied Biosystems) and 500 μM forward and reverse primers. Real-time PCR was performed in an ABI PRISM 7500 sequence detection system (Applied Biosystems) under the following thermal cycling conditions: 10 min at 95°C followed by a total of 40 cycles of 30 s at 95°C, 30s min at 65°C and 40s at 72°C. For qRT-PCR, relative transcript abundance was calculated and normalized with respect to *ACTIN *transcript levels. All calculations and analyses were performed using ABI 7500 Software v2.0.1 (Applied biosystems) and the 2^-ΔΔCt ^method [92]. Amplification efficiency (0.92 to 1.01) for the primer sets was determined by amplification of a cDNA dilution series (1:5). Specificity of the RT-PCR products was followed by a melting curve analysis with continual fluorescence data acquisition during the 65-95°C melt.

### ROS Detection

For H_2_0_2 _localization, the DAB staining method was performed as described by Orozco and Ryan (1999, [93]) and the stained *U. gibba *plants were cleared by the method described by Malamy and Benfey (1997, [94]) and analyzed with an SZH10 stereomicroscope (Olympus).

## List of Abbreviations

UT: Unique Transcripts; PEG: Preferentially expressed genes; TrpL: cDNA library generated from *U. gibba *traps; FlwL: cDNA library generated from *U. gibba *inflorescences; ShtL: cDNA library generated from *U. gibba *shoots (stem/leaves); StsL: cDNA library generated from whole plants subjected to multiple physiological stress condition; ROS: Reactive Oxygen Species

## Authors' contributions

EIL performed assembling, annotation, database construction, statistical analysis and manuscript writing. VAA contributed to data analysis, phylogenetic analysis, drafting and editing of the manuscript. CAPT and MJOE carried out RNA extractions and cDNA synthesis. CAPT also participated in the ROS staining experiments. FMZH performed qRT-PCR experiments. AHE and LHE conceived of the project and were responsible for directing all of the research activities, and also have assisted in the writing of the manuscript. All authors have read and approved the final submitted version of the manuscript.

## Supplementary Material

Additional file 1 Table S1 - *U. gibba *UT annotated by searching for sequences similarities using BLASTx (e-value ≤10-05 and a bit score ≥ 45) and proteins data bases from *Arabidopsis thaliana, Populus trichocarpa, Ricinus communis, Vitis vinifera *(dicotyledon plants), *Oryza sativa, Sorghum bicolor *(monocotyledon plants), *Physcomitrella patens *(moss), *Chlamydomonas reinhardtii*, and *Ostreococcus lucimarinuas *(green algae) Click here for file

Additional file 2 **Figure S1 - Metabolic pathways represented in the *U. gibba *unique transcripts (UT) set**. **(A) **Global metabolism map constructed combining existing pathway maps and corresponding genes referenced in the KEGG database for Plants (black lines). **(B) **Global metabolism map represented by the *U. gibba *UT (blue lines). **(C) **Overlap comparison of the KEGG metabolic global map of flowering plants to the metabolic map represented in *U. gibba *UT. Click here for file

Additional file 3 **Table S2 - Gene numbers comparison of TF families members indentified in *U. gibba *with some vascular plants (*Arabidosis thaliana, Populus trichocarpa *and *Oryza sativa *(indica/japonica))**. Click here for file

Additional file 4 **Table S3 - Expression profile matrix of *U. gibba *genes.** Reads-counts (raw and normalized) for every specific-transcriptome (FlwL; Inflorescense, ShtL; shoot, TrpL; Traps and StsL; stressed plants) merged into each one of the 16,551 contigs. Click here for file

Additional file 5 **Table S4 - Preferentially Expressed Genes (PEG) in *U. gibba *organ specific**. Click here for file

Additional file 6 **Table S5 - Non organ-specific genes of *U. gibba *plants (Ubiquitous)**. Click here for file

Additional file 7 **Table S6 - Stress responsive genes identified in *U. gibba *plants**. Click here for file

Additional file 8 **Table S7 - Functional categories over-represented in physiological stress conditions (StsL), inflorescense (FlwL), shoot (ShtL) and traps (TrpL) *U. gibba *PEGs**. Categorization of *U. gibba *UT was obtained according to the MIPS classification using FunCat database. Click here for file

Additional file 9 **Figure S2 - Validation of PEGs by qRT-PCR**. Expression patterns of APETALA1, APETALA3, PISTILLATA, AGAMOUS, SEPALATA3, CLAVATA1, MYB21, MYB24, RBCS-1B, SBPase, α-glucosidase, ß-hexosaminidase, aminopeptidase, acid phosphatase and ß-glucosidase are presented as obtained with 454 sequencing **(A) **and qRT-PCR **(B)**. Correlation of expression levels as obtained from 454 sequencing with those obtained with qRT-PCR **(C)**. Click here for file

Additional file 10 **Table S8 - *U. gibba *UT annotated as homologous *Arabidopsis *Pi transporters**. Click here for file

Additional file 11 **Table S9 - *U. gibba *UT annotated as homologous Arabidopsis genes involved in Nitrate assimilation pathway**. Click here for file

Additional file 12 **Figure S3 - Alignment of aa sequences of *Arabidopsis *α- and γ- Tonoplast Intrinsic Proteins (AT3G16240, AT2G36830, respectively) previously characterized as urea transporters **[63]**and homologous *U. gibba *UT identified as shoot (stem/leaves) PEGs**. 'NPA' boxes, which are typical for plant TIPs, are highlighted by a box with discontinuous red line. Click here for file

Additional file 13 **Figure S4 - Fraction of *U. gibba *LSC chloroplast region used in phylogenetic analysis**. **(A) **Plastid genome comparison of two closely related species (*Solanum lycopersicum *and *Jasminum nudiflorum*) and the homologous region of the *Utricularia. gibba *LSC region. **(B) **Plastid genes identified in this region. **(C) **Non-ambiguously-aligned region (blocks) used in phylogenetic analysis. Click here for file

Additional file 14 **Figure S5 - *U. gibba *mitochondrial region used in phylogenetic analysis**. **(A) **Mitochondrial genome comparison of *Arabidopsis thaliana, Brassica napus, Carica papaya, Nicotianan tabacum *and *Vitis vinifera *and the homologous region from *Utricularia gibba*. **(B) **Mitochondrial genes identified in this region. **(C) **Non-ambiguously-aligned region (blocks) used in phylogenetic analysis. Click here for file

Additional file 15 **Table S10 - *U. gibba *UT annotated as homologous *Arabidopsis *genes involved in DNA repair system**. Click here for file

Additional file 16 **Table S11 - *U. gibba *UT annotated as homologous *Arabidopsis *genes involved in ROS detoxification system**. Click here for file
