# Supplementary figures and images for: Transcriptomics and molecular evolutionary rate analysis of the bladderwort (Utricularia), a carnivorous plant with a minimal genome
Source: BMC Plant Biol. 2011 Jun 3;11:101. doi: 10.1186/1471-2229-11-101 (PMC3141634; doi:10.1186/1471-2229-11-101)

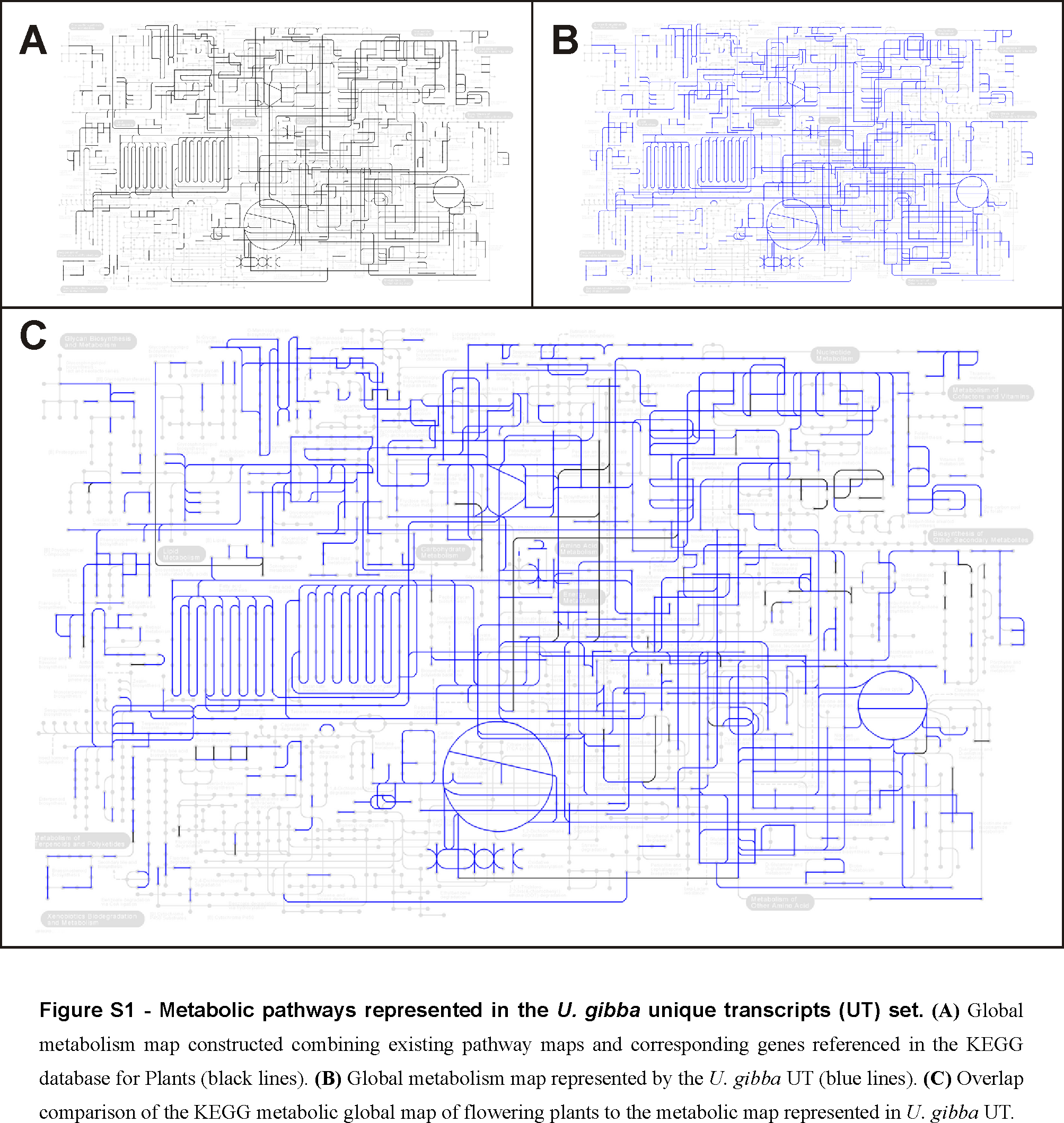

Supplement: Additional file 2 — Figure S1 - Metabolic pathways represented in the U. gibba unique transcripts (UT) set. (A) Global metabolism map constructed combining existing pathway maps and corresponding genes referenced in the KEGG database for Plants (black lines). (B) Global metabolism map represented by the U. gibba UT (blue lines). (C) Overlap comparison of the KEGG metabolic global map of flowering plants to the metabolic map represented in U. gibba UT. [file 1471-2229-11-101-S2.TIFF]

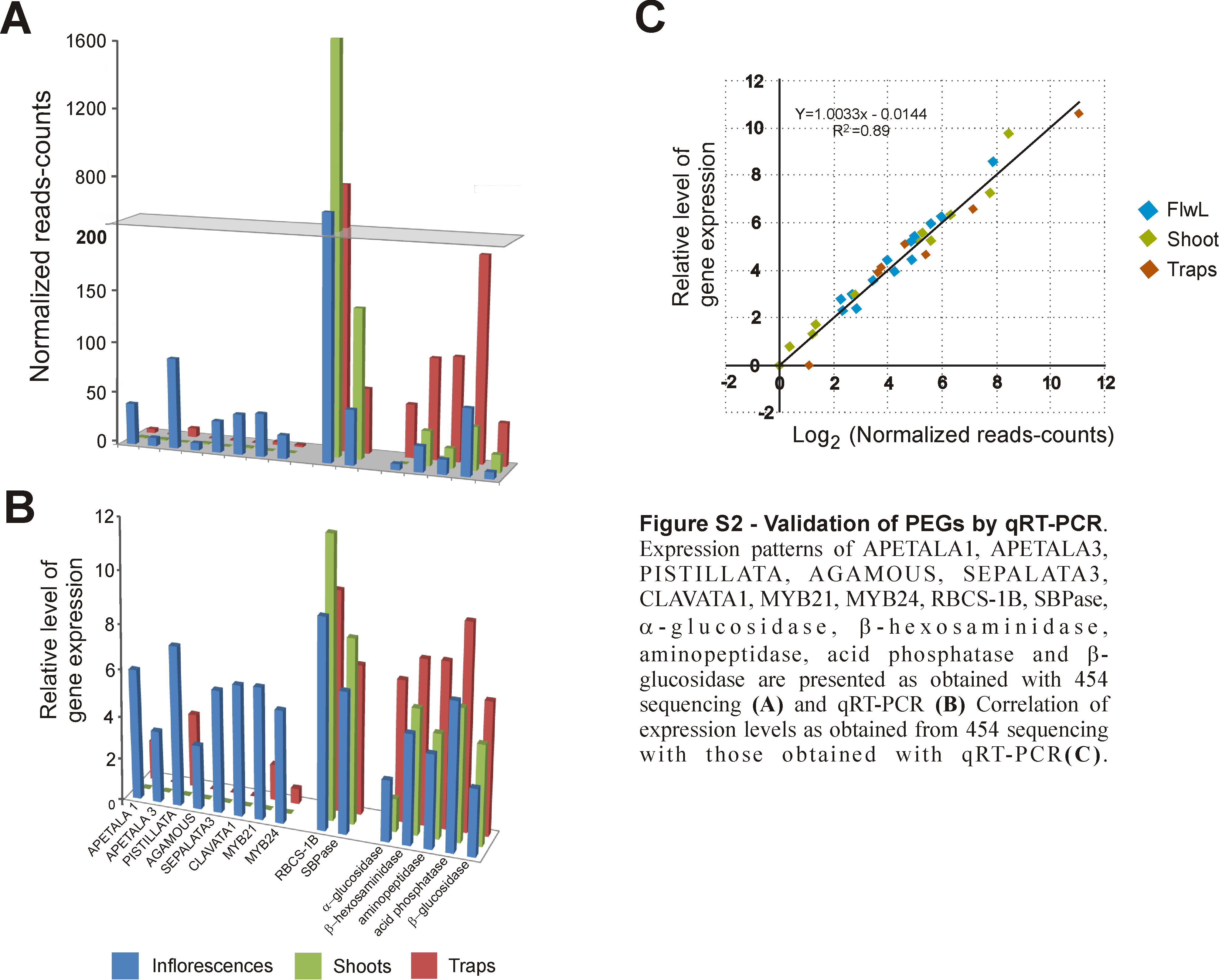

Supplement: Additional file 9 — Figure S2 - Validation of PEGs by qRT-PCR. Expression patterns of APETALA1, APETALA3, PISTILLATA, AGAMOUS, SEPALATA3, CLAVATA1, MYB21, MYB24, RBCS-1B, SBPase, α-glucosidase, ß-hexosaminidase, aminopeptidase, acid phosphatase and ß-glucosidase are presented as obtained with 454 sequencing (A) and qRT-PCR (B). Correlation of expression levels as obtained from 454 sequencing with those obtained with qRT-PCR (C). [file 1471-2229-11-101-S9.TIFF]

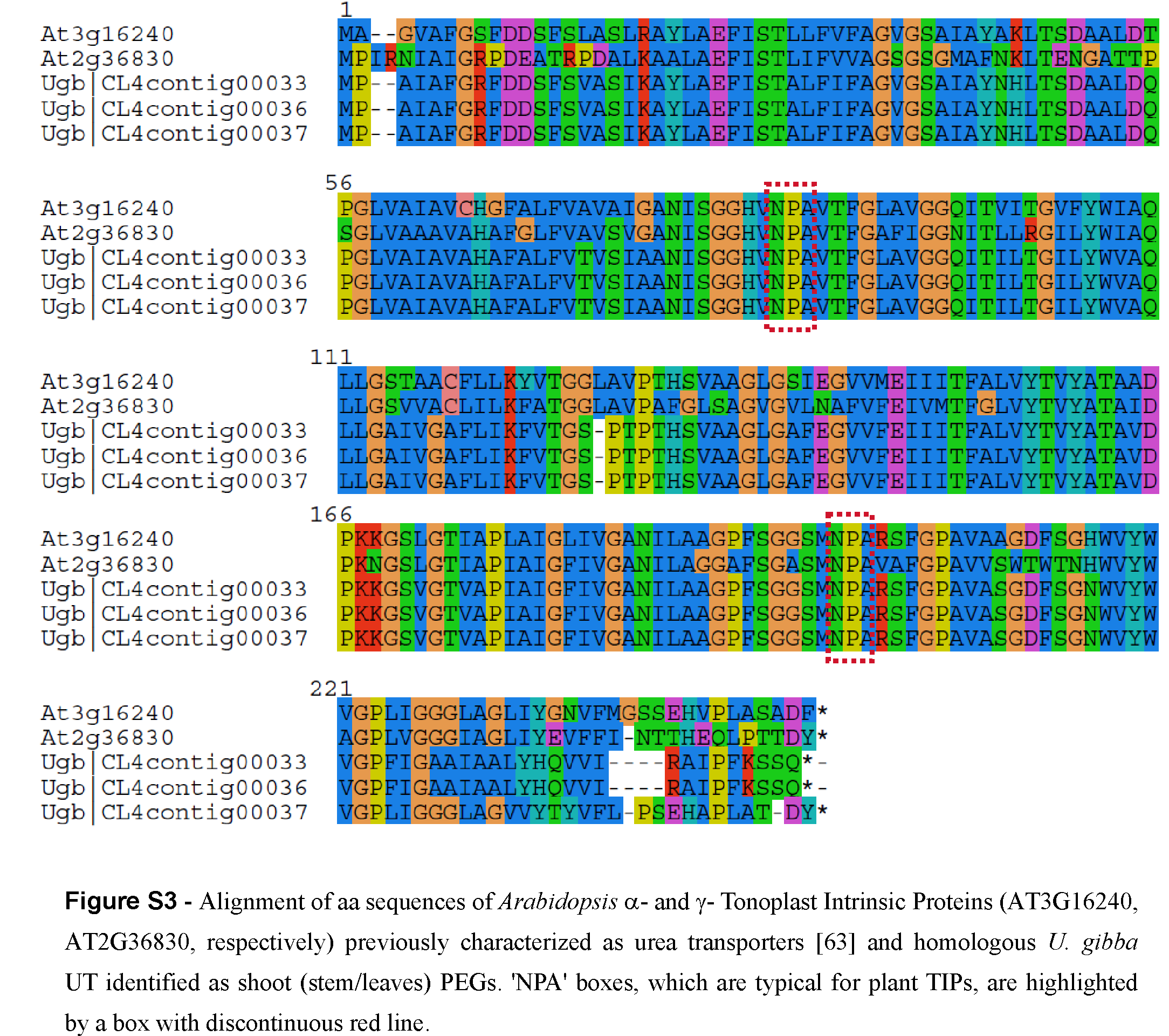

Supplement: Additional file 12 — Figure S3 - Alignment of aa sequences of Arabidopsis α- and γ- Tonoplast Intrinsic Proteins (AT3G16240, AT2G36830, respectively) previously characterized as urea transporters [63]and homologous U. gibba UT identified as shoot (stem/leaves) PEGs. 'NPA' boxes, which are typical for plant TIPs, are highlighted by a box with discontinuous red line. [file 1471-2229-11-101-S12.TIFF]

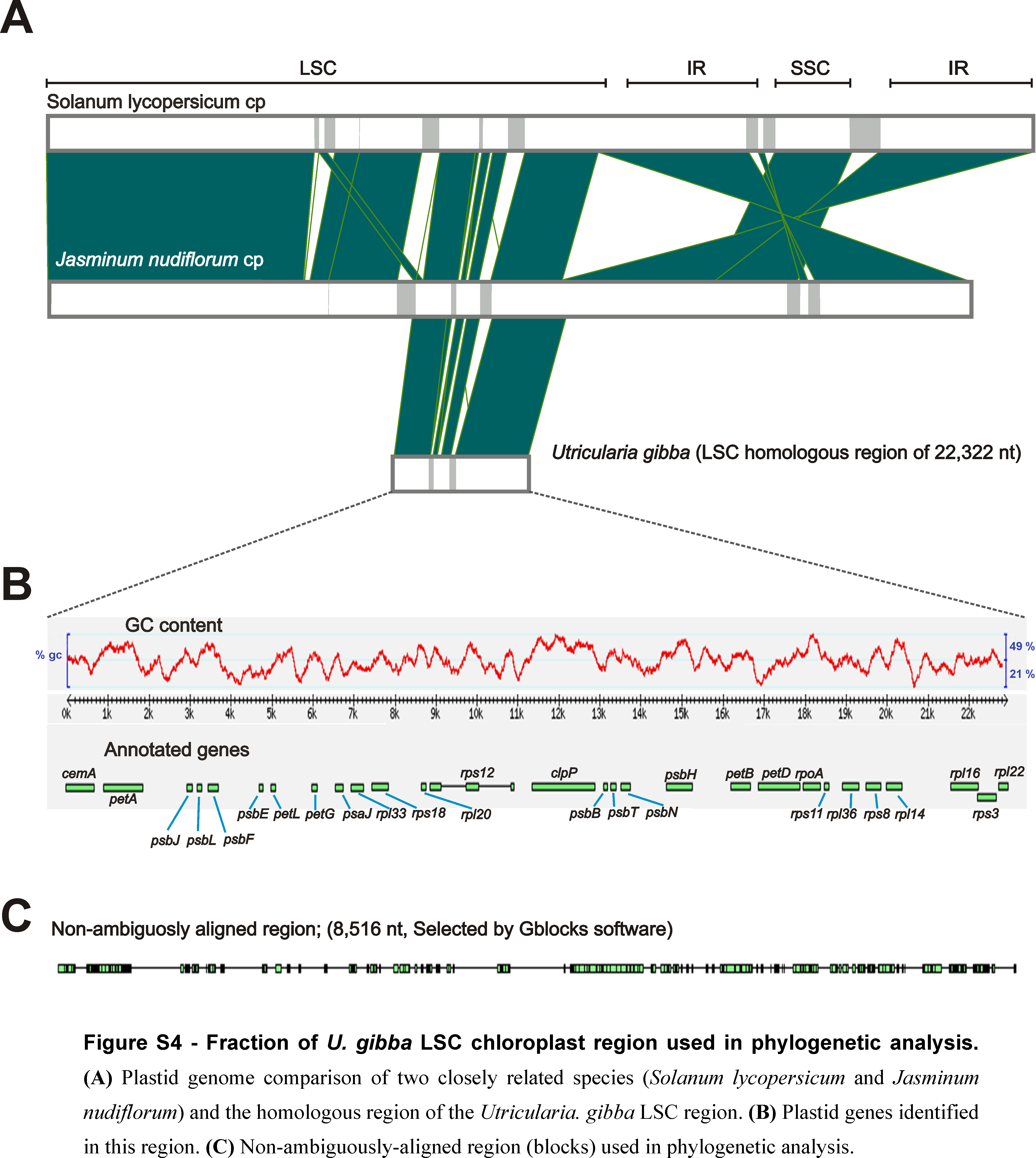

Supplement: Additional file 13 — Figure S4 - Fraction of U. gibba LSC chloroplast region used in phylogenetic analysis. (A) Plastid genome comparison of two closely related species (Solanum lycopersicum and Jasminum nudiflorum) and the homologous region of the Utricularia. gibba LSC region. (B) Plastid genes identified in this region. (C) Non-ambiguously-aligned region (blocks) used in phylogenetic analysis. [file 1471-2229-11-101-S13.TIFF]

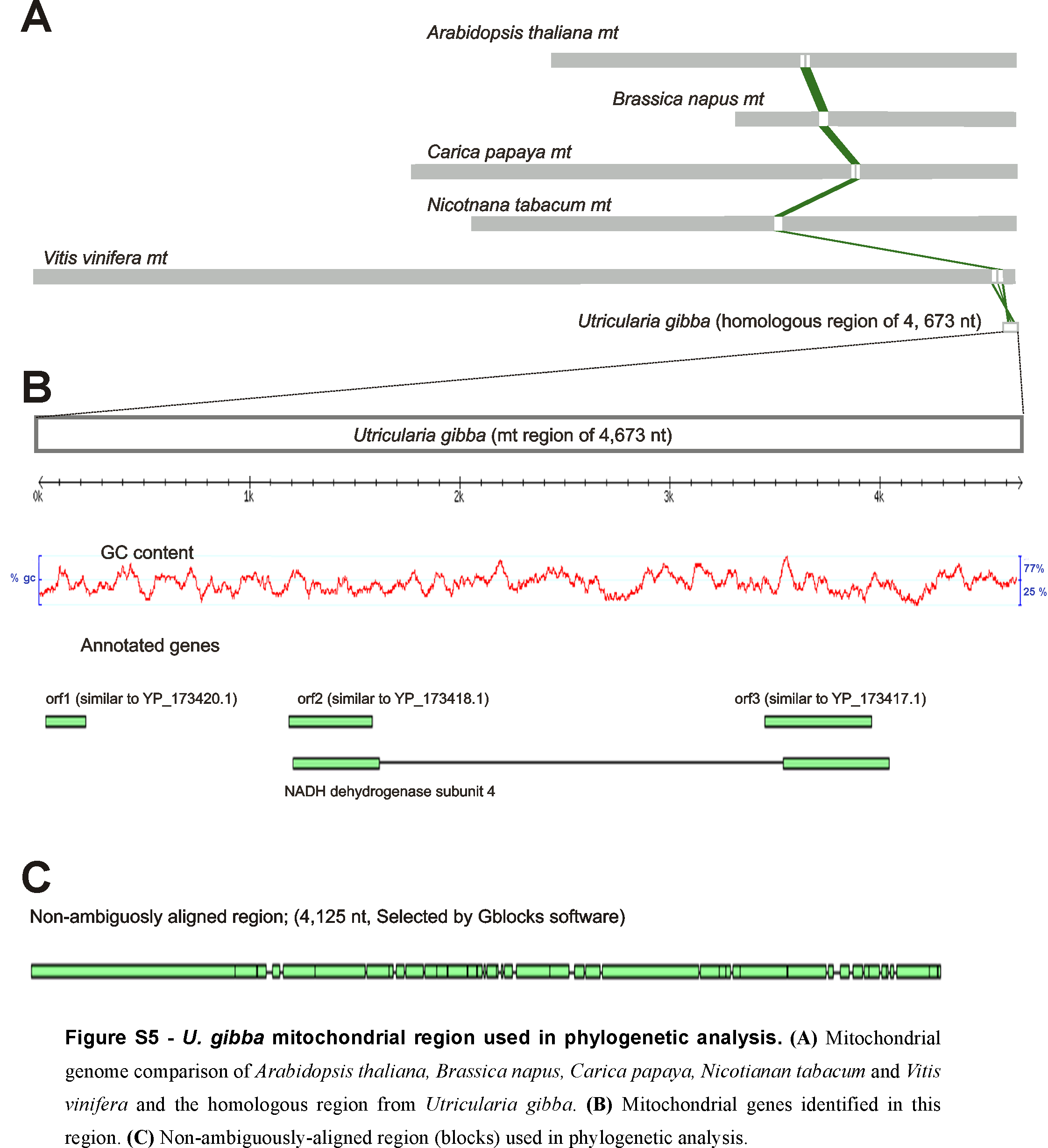

Supplement: Additional file 14 — Figure S5 - U. gibba mitochondrial region used in phylogenetic analysis. (A) Mitochondrial genome comparison of Arabidopsis thaliana, Brassica napus, Carica papaya, Nicotianan tabacum and Vitis vinifera and the homologous region from Utricularia gibba. (B) Mitochondrial genes identified in this region. (C) Non-ambiguously-aligned region (blocks) used in phylogenetic analysis. [file 1471-2229-11-101-S14.TIFF]
